# Non-coding RNAs as emerging players in *Leishmania* development and host-parasite interactions

**DOI:** 10.3389/fcimb.2025.1682470

**Published:** 2025-11-04

**Authors:** Eyson Quiceno, Zemfira N. Karamysheva

**Affiliations:** Department of Cell Biology and Biochemistry, Texas Tech University Health Science Center, Lubbock, TX, United States

**Keywords:** non-coding RNA, Leishmania, post-transcriptional regulation, translational control, immune response, host-parasite interactions

## 1 Introduction

Leishmaniasis is a neglected disease, widespread throughout the world. It represents a major global health challenge due to its economic and social implications. It is caused by protozoan parasites of the genus *Leishmania* (Mann et al., 2021; Mathison and Bradley Benjamin, 2022). Unlike most eukaryotes, *Leishmania* has an atypical genome, characterized by its high plasticity (Thomas et al., 2009; Rogers et al., 2011; Iantorno et al., 2017; Glans et al., 2021), the absence of introns, polycistronic constitutive transcription of its genes and lack of gene regulation at the transcriptional level (Martinez-Calvillo et al., 2003; Ivens et al., 2005; Peacock et al., 2007; Rogers et al., 2011). Because of this, the regulation of gene expression in *Leishmania* and trypanosomatids in general occurs at the post-transcriptional level. Recent studies have found that non-coding RNAs (ncRNAs) play an important role in these regulatory mechanisms in trypanosomatids, however, the precise function and mechanisms associated with them are poorly understood (Rajan et al., 2020; Fort et al., 2022; Guegan et al., 2022; Espada et al., 2025; Quilles et al., 2025).

In general, ncRNAs are a class of RNA transcripts that are not translated into proteins but serve essential regulatory functions in a variety of biological processes. ncRNA are typically categorized based on length or functions. Based on length, they are classified into small ncRNAs (<200 nucleotides) and long ncRNAs (>200 nucleotides) (Quinn and Chang, 2016; Zhang et al., 2019; Chen and Kim, 2024; Jouravleva and Zamore, 2025). Based on function, they are divided into two major categories: 1) housekeeping ncRNAs (ribosomal RNAs (rRNAs), transfer RNAs (tRNAs), small nuclear RNAs (snRNAs), small nucleolar RNAs (snoRNAs) and telomerase RNA (TERC); these ncRNAs are ubiquitously expressed and participate in fundamental cellular activities and 2) regulatory ncRNAs (microRNAs (miRNAs), small interfering RNAs (siRNAs), PIWI-interacting RNAs (piRNAs), tRNA-derived fragments (tRFs), and tRNA halves (tiRNAs), enhancer RNAs (eRNAs), long non-coding RNAs (lncRNAs), and circular RNAs (circRNAs)); these are involved in fine-tuning gene expression at multiple levels — epigenetic, transcriptional, and post-transcriptional (Pasquinelli and Ruvkun, 2002; Ambros, 2003; Zhang et al., 2019; Chen and Kim, 2024).

Similar to other eukaryotes, *Leishmania* parasites carry same types of housekeeping ncRNAs. A diverse repertoire of regulatory ncRNAs has been identified across various *Leishmania* species including siRNAs (Atayde et al., 2013; Lambertz et al., 2015; Brettmann et al., 2016), tRNA- and rRNA-derived small RNAs (Lambertz et al., 2015; Kusakisako et al., 2023), snoRNAs (Liang et al., 2007; Saxena et al., 2007; Eliaz et al., 2015; Piel et al., 2022; Rajan et al., 2024), and lncRNAs (Dumas et al., 2006; Emond-Rheault et al., 2025; Espada et al., 2025). Although many *Leishmania* species lost the canonical RNA interference (RNAi) pathway, however, several species in the *Viannia* subgenus (*Leishmania braziliensis* and *Leishmania guyanensis)* (Lye et al., 2022) retain a functional RNAi machinery capable of producing siRNAs (Atayde et al., 2013; Brettmann et al., 2016). Importantly, while canonical miRNAs have not been identified in *Leishmania*, some *in silico* studies have proposed the existence of miRNA-like molecules with potential regulatory function (Sahoo et al., 2014; Nimsarkar et al., 2020; Martinez-Hernandez et al., 2025). However, these findings remain speculative and require *in vitro* validation before any definitive conclusions can be drawn. Complementing these predictions, various RNA-seq datasets have revealed a wide range of ncRNAs encoded in the *Leishmania* genome. In many cases these transcripts remain functionally uncharacterized; they have been identified in species such as *L. braziliensis* (Torres et al., 2017; Ruy et al., 2019; Martinez-Hernandez et al., 2025; Quilles et al., 2025), *L. amazonensis* (Aoki et al., 2017; Goes et al., 2023), *L. major* (Liang et al., 2007; Eliaz et al., 2015; Freitas Castro et al., 2017; Rajan et al., 2024; Martinez-Hernandez et al., 2025), *L. donovani* (Saxena et al., 2007; Freitas Castro et al., 2017; Piel et al., 2022; Martinez-Hernandez et al., 2025), *L. infantum* (Dumas et al., 2006; Emond-Rheault et al., 2025), *L. mexicana* (Kalesh et al., 2022).

Across a wide range of organisms, ncRNAs have been implicated in mRNA processing, mRNA stability and emerge as key players in a variety of regulatory processes, such as DNA replication, chromosome maintenance, transcriptional regulation, translation, protein stability, the translocation of regulatory proteins and host-parasite interactions (Freitas Castro et al., 2017). Understanding the role and mechanisms of ncRNAs actions in *Leishmania* and host may lead to new avenues in the search for strategies to control leishmaniasis.

## 2 Post-transcriptional regulation and translational control

**Figure 1:**
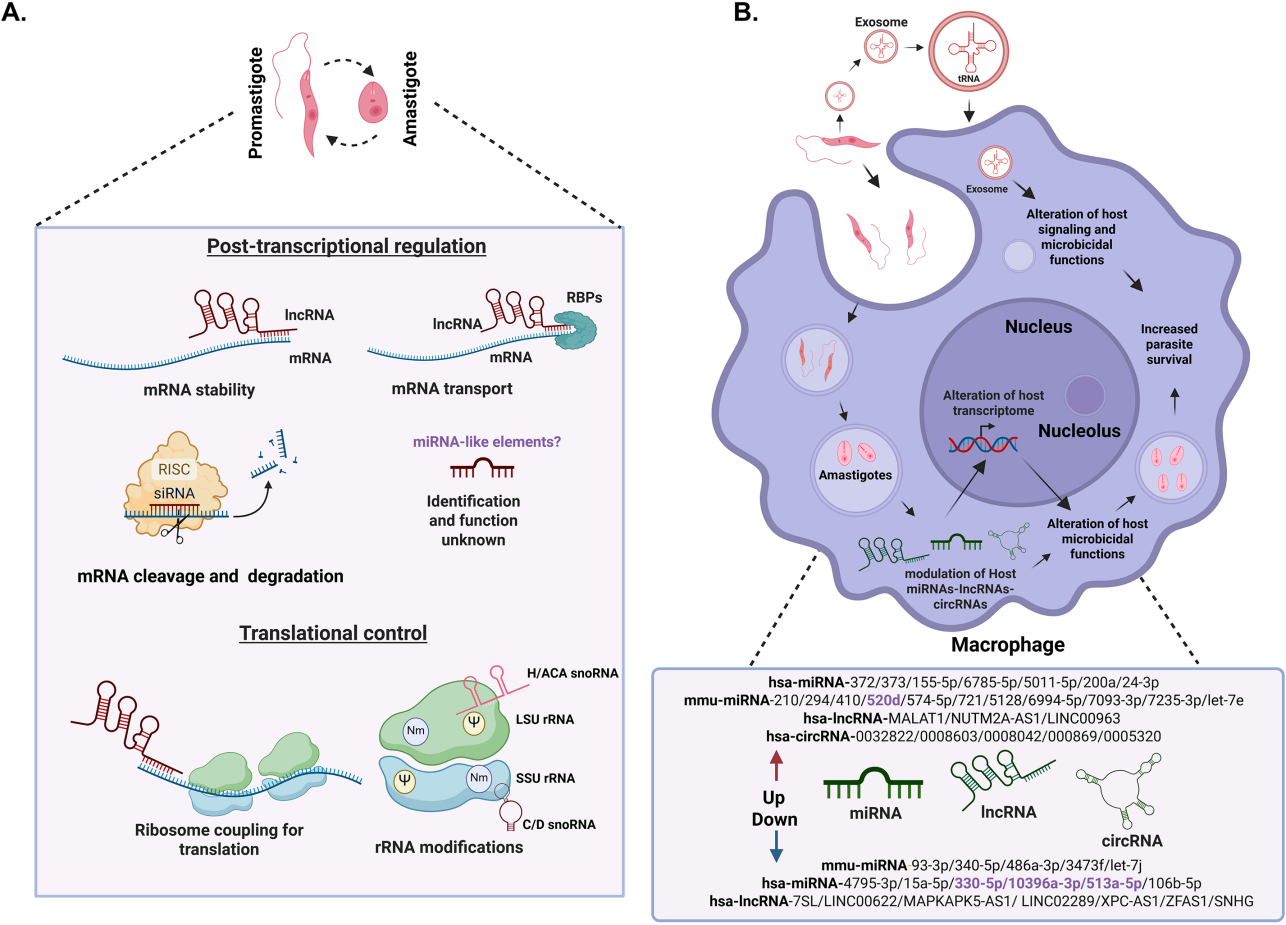
The role of ncRNAs in *Leishmania* parasites and host cells. **(A)**. Modulation of ncRNAs in *Leishmania* parasites during development: we show the role of ncRNAs in different steps of gene expression in *Leishmania* promastigotes and amastigotes. At the post-transcriptional level, expression level is regulated through mRNA stability, translation, transport and degradation, as well as the possible presence of microRNA-like elements. Ribosome RNA (rRNA) modifications such as 2’-O-methylation (Nm) (Purple circle) and pseudouridylation (Ψ) (Yellow circle) by snoRNAs modulate mRNA translation. These modifications are essential for the parasite, as they allow it to adapt and survive in different environments through modifications of ribosomal RNA (rRNA), influencing ribosome biogenesis and gene expression regulation at multiple levels. **(B)** This figure shows schematic changes in a macrophage during the phagocytosis of *Leishmania* and is not to the scale. Modulation of host ncRNAs during *Leishmania* infection. *Leishmania* can mainly modulate micro RNAs (miRNAs), long non-coding RNAs (lncRNAs) and circular RNAs (circRNAs) in the host. These changes lead to the alterations in the host transcriptome, immune response evasion and increased parasite survival. Host transcripts are shown in green and *Leishmania* parasite transcripts in red. The ncRNAs in black correspond to those validated with RT-qPCR, while in purple correspond to those that have not yet been validated. In the description of ncRNAs, “hsa” refers to ncRNAs from humans (*Homo sapiens*) while “mmu” refers to ncRNAs from mice (*Mus musculus*).Biorender software was used to create this figure under an academic license.

*Leishmania* parasites are characterized by the absence of classic genetic control at the transcriptional level, therefore, one of the main roles of ncRNAs could be at the post-transcriptional level (**Figure 1A**), through regulation of mRNA stability, processing, transport, and degradation; these processes at the post-transcriptional level have been seen in other protozoa (Li et al., 2020; Simantov et al., 2022). Recent evidence suggests a significant presence of regulatory ncRNAs derived from untranslated regions (UTRs) of mRNAs (Freitas Castro et al., 2017). RNA-Binding Proteins (RBPs) are central to post-transcriptional regulation (Glisovic et al., 2008). Over 2,400 RBPs, including non-poly(A)-binding proteins, form complexes with ncRNAs, suggesting roles in RNA transport and stability in *L. mexicana* (Kalesh et al., 2022).

In the absence of transcriptional regulation translational control plays a crucial role in *Leishmania* gene expression supporting survival and adaptation to dramatically different environments during change of host (Jaramillo et al., 2011; Cloutier et al., 2012; Karamysheva et al., 2020; Gutierrez Guarnizo et al., 2023; Rodriguez-Almonacid et al., 2023). In eukaryotes ncRNAs play direct roles in modulating protein synthesis, either by interacting with ribosomes, regulating the availability of mRNAs for translation or governing modifications of ribosomal proteins (Godet et al., 2024). rRNAs facilitate the peptidyl transfer reaction during protein synthesis (Moore and Steitz, 2011). Recent studies support importance of rRNA modifications by snoRNAs in *L. major* (Eliaz et al., 2015). snoRNAs are organized in gene clusters containing both C/D and H/ACA types, guiding rRNA modifications like 2’-O-methylation (Nm) and pseudouridylation (Eliaz et al., 2015; Rajan et al., 2024). These modifications occur in conserved rRNA domains and are critical for rRNA maturation, stability and mRNA translation.

In *L. infantum*, a class of lncRNAs (300–600 nucleotides) was mainly identified in amastigotes, showing a preferential association with the small ribosomal subunit (40S) (Dumas et al., 2006). These findings indicate a possible role in regulation at the translation level, although a direct effect on translational initiation has not been demonstrated. In *L. braziliensis*, the lncRNA45 was functionally characterized, demonstrating possible roles in RNA processing and modulation of translation rates, either enhancing or impairing protein synthesis (Espada et al., 2025). Additionally, a small ncRNA called ncRNA97, was found to be preferentially expressed in the amastigote form of *L. braziliensis* (Quilles et al., 2025). This ncRNA modulates gene expression through the control of the stability of the mRNAs that are involved in metacyclogenesis and responses to nutritional stress, indicating a role in developmental adaptation.

These examples underscore the intricate and multifaceted roles of ncRNAs in regulating gene expression in *Leishmania* parasites. However, the number of examples is limited, so the specific functions and mechanisms are still areas to be explored.

## 3 Modulation of host ncRNAs by *Leishmania* parasites

*Leishmania*, being an intracellular pathogen, has machinery that allows it to adapt and survive the hostile environment within the hosts. One of the main mechanisms of *Leishmania* to alter the host’s responses favoring parasite survival involves host transcriptome remodeling that includes modulating the expression of both coding RNAs and ncRNAs such as miRNAs (Lemaire et al., 2013; Pandey et al., 2016; Singh et al., 2016; Muxel et al., 2017; Kumar et al., 2018; Muxel et al., 2018; Kumar et al., 2020; Acuña et al., 2022; Lago et al., 2023; Fernandes et al., 2024; Hadifar et al., 2024; Tabrez et al., 2024; Akand et al., 2025; Atri et al., 2025; Masoudsinaki et al., 2025; Roy et al., 2025), lncRNAs (Misra et al., 2005; Maruyama et al., 2022; Fernandes et al., 2023; Li et al., 2023; Hadifar et al., 2024; Sirekbasan and Gurkok-Tan, 2025) and circRNAs levels (Li et al., 2022; Alizadeh et al., 2025), (**Figure 1B**). Interestingly, 30% of differentially expressed transcripts in infected macrophages correspond to lncRNAs supporting their importance to control macrophage function during infection (Fernandes et al., 2023).

*Leishmania* modulates host immune responses via alteration of host ncRNA expression profiles, affecting processes such as apoptosis, phagocytosis, and immune signaling (Fernandes et al., 2023; Scaramele et al., 2024; Tabrez et al., 2024; Akand et al., 2025). *L. donovani* infection in CD4+ T cells upregulates certain miRNAs (miR-6994-5p, miR-5128, miR-7093-3p, miR-574-5p and miR-7235) which interferes with the expression of the pro-inflammatory cytokine IFN-γ (Kumar et al., 2020). Moreover, the downregulation of miR-340-5p, miR-93-3p, let 7j, 486a-3p and miR-3473f promotes the differentiation of macrophages towards a Th2 phenotype, favoring the survival of the parasite. In *L. braziliensis* the upregulation of miR-2940-3p and miR-5100 caused suppression of TNF and NF-κB pathways, reducing inflammatory responses (Lago et al., 2023). *L. amazonensis* parasites are able to change the TLR signaling pathways by modulating the expression level of miRNA-let-7e (Muxel et al., 2018). The upregulation of this miRNA decreases the inflammatory response of host cells. Also, *L. amazonensis* induces an upregulation of miR-294 and miR-721 in macrophages (Muxel et al., 2017). This upregulation causes a repression of inducible nitric oxide synthase (NOS2) leading to reduced production of nitric oxide and subsequent decrease in the capability of the macrophage to kill the parasites. *L donovani* and *L. major* causes an upregulation of miR-210 in macrophages under hypoxic conditions, leading to downregulation of pro-inflammatory cytokines and enhancing parasite survival (Lemaire et al., 2013; Kumar et al., 2018).

*L. infantum* infection has been shown to alter the expression of numerous lncRNAs in human neutrophils, leading to the impairment of key antimicrobial responses such as phagocytosis and nitric oxide production (Scaramele et al., 2024). This contributes to immune evasion and the survival of the parasite. In THP-1 macrophages, the infection with *L. amazonensis*, *L. braziliensis*, and *L. infantum* led to a differential expression of different host lncRNAs upon infection suggesting a mechanism by which *Leishmania* can control macrophages and evade the immune response (Fernandes et al., 2023). A test performed on peripheral blood from patients infected with visceral leishmaniasis cured patients; asymptomatic infected individuals and healthy controls showed that *L. infantum* alters the expression of host lncRNAs (Maruyama et al., 2022). These lncRNAs are co-expressed with immune-related protein-coding genes and may regulate immune pathways, potentially influencing the host’s ability to respond to infection. *Leishmania* infection of macrophages leads to downregulation of host lncRNA 7SL RNA, an essential component of the signal recognition particle (SRP), which is responsible for targeting newly synthesized proteins to the endoplasmic reticulum (ER). This generates a downregulation of protein targeting and secretion altering trafficking of immune effectors and antigen presentation, favoring parasite persistence (Misra et al., 2005).

*Leishmania* influences host cellular metabolism to create a more favorable environment for its survival. miR-210 has been linked to altered L-arginine metabolism, leading to a reduction in nitric acid production. Certain miRNAs (miR-372/373/520d family) are upregulated in human macrophages during infection with *L. amazonensis*, leading to changes in arginine metabolism and increased polyamine production, which supports parasite survival (Fernandes et al., 2024). Inhibiting these miRNAs reduces parasite survival. During infection of bone marrow-derived macrophages with *L. amazonensis* the miRNAs miR-294 and miR-410 were upregulated; these miRNAs can interfere with the production of L-arginine and the immune response in the macrophages (Acuña et al., 2022). *L. donovani* also modulates host miRNAs that regulate cholesterol and sphingolipid biosynthesis which are crucial for the parasite’s survival (Tabrez et al., 2024; Akand et al., 2025). While the functional impact of lnRNAs and miRNAs is only beginning to be uncovered, host circRNAs have emerged as another relevant class of ncRNAs in leishmaniasis. A recent study found a large number of circRNAs differentially expressed in the serum of patients with leishmaniasis compared with healthy controls (Li et al., 2022) while in THP-1 cells infected with *L. tropica and L. infantum* distinct circRNAs profiles were found depending on the parasite strain (Alizadeh et al., 2025).

In addition to directly modulating host ncRNAs, *Leishmania* also influences the host environment through the secretion of extracellular vesicles, particularly exosomes (**Figure 1B**). During infection, those exosomes can modulate the host immune response (Silverman and Reiner, 2011; Peng et al., 2022; Sharma and Singh, 2025). tRFs and other small RNAs have been detected in exosomes secreted by *Leishmania*, suggesting a role in intercellular communication and possibly in the modulation of host translation (Lambertz et al., 2015; Kusakisako et al., 2023). These vesicles can modulate immune responses in different ways. It has been found that the parasite is able to release exosomes in sand flies and host cells, which can stimulate an inflammatory response leading to exacerbated cutaneous leishmaniasis (da Silva Lira Filho et al., 2022). *In vivo* studies have demonstrated that treatment of mice with *L. donovani* exosomes prior to challenge with the parasite exacerbates infection, promotes IL-10 and inhibits TNF-α production (Silverman et al., 2010). These findings indicate that *Leishmania* exosomes, with their ncRNA cargo, are predominantly immunosuppressive and play a significant role in shaping the host immune response to favor parasite survival.

Together, the modulation of host miRNAs, lncRNAs and circRNAs as well as the production of exosomes in the parasite uncovers the strategy by which *Leishmania* manipulates host immune responses and cellular functions for its own benefit. While progress has been made in studying how the parasite alters host ncRNAs, the possible functions of *Leishmania’*s own ncRNAs (beyond those secreted into exosomes) remain largely unexplored. Understanding these mechanisms of host-parasite interactions would allow to identify new therapeutic targets.

## 4 Discussion

Although recent studies have expanded our understanding of the roles of ncRNAs in *Leishmania*, their precise functions and mechanisms remain unclear, largely because most findings are based on transcriptomic descriptions rather than functional validation. Current solid evidence indicates that host miRNA modulation plays a major role in the survival of the parasite, mainly through the modulation of effective host immune response. However, growing evidence shows that other host ncRNAs, including lncRNAs and most recently circRNAs are also altered during infection and may contribute to parasite survival (**Figure 1B**).

It is clear that parasite is able to modulate host ncRNAs, however, the role of the parasite’s own ncRNAs in this modulation is poorly understood. This represents a significant gap and an opportunity for future research. To move beyond simple characterization, we must fully understand the mechanism of their action and what they target in the host and parasite. Advanced tools such as single-cell transcriptomics, polysome profiling and CRISPR-based gene editing could help to validate their role and functions in both the host and parasites.

This research can open new avenues for combating leishmaniasis. ncRNAs could serve as therapeutic targets, be used as biomarkers for diagnosis and prognosis, or even be developed as a tool to restore or enhance the host’s immune response. The growing body of evidence highlights the essential roles of ncRNAs in parasite biology and host-pathogen interactions. As the field advances, ncRNAs may deepen our understanding of *Leishmania* pathogenesis.
